# Neurodevelopmental disturbances in schizophrenia: evidence from genetic and environmental factors

**DOI:** 10.1007/s00702-022-02567-5

**Published:** 2022-11-12

**Authors:** Andrea Schmitt, Peter Falkai, Sergi Papiol

**Affiliations:** 1 Department of Psychiatry and Psychotherapy, University Hospital, LMU Munich, Nußbaumstr. 7, 80336 Munich, Germany; 2 Laboratory of Neuroscience (LIM27), Institute of Psychiatry, University of São Paulo, São Paulo, Brazil; 3 Max Planck Institute of Psychiatry, Kraepelinstr. 2-10, Munich, Germany; 4 Institute of Psychiatric Phenomics and Genomics (IPPG), University Hospital, LMU Munich, Munich, Germany

**Keywords:** Schizophrenia, Neurodevelopment, Risk genes, Environmental factors, Connectivity, Synaptic plasticity, Neuron, Oligodendrocyte

## Abstract

Since more than 3 decades, schizophrenia (SZ) has been regarded as a neurodevelopmental disorder. The neurodevelopmental hypothesis proposes that SZ is associated with genetic and environmental risk factors, which influence connectivity in neuronal circuits during vulnerable developmental periods. We carried out a non-systematic review of genetic/environmental factors that increase SZ risk in light of its neurodevelopmental hypothesis. We also reviewed the potential impact of SZ-related environmental and genetic risk factors on grey and white matter pathology and brain function based on magnetic resonance imaging and post-mortem studies. Finally, we reviewed studies that have used patient-derived neuronal models to gain knowledge of the role of genetic and environmental factors in early developmental stages. Taken together, these studies indicate that a variety of environmental factors may interact with genetic risk factors during the pre- or postnatal period and/or during adolescence to induce symptoms of SZ in early adulthood. These risk factors induce disturbances of macro- and microconnectivity in brain regions involving the prefrontal, temporal and parietal cortices and the hippocampus. On the molecular and cellular level, a disturbed synaptic plasticity, loss of oligodendrocytes and impaired myelination have been shown in brain regions of SZ patients. These cellular/histological phenotypes are related to environmental risk factors such as obstetric complications, maternal infections and childhood trauma and genetic risk factors identified in recent genome-wide association studies. SZ-related genetic risk may contribute to active processes interfering with synaptic plasticity in the adult brain. Advances in stem cell technologies are providing promising mechanistic insights into how SZ risk factors impact the developing brain. Further research is needed to understand the timing of the different complex biological processes taking place as a result of the interplay between genetic and environmental factors.

## Introduction

Mental disorders, including schizophrenia (SZ), are the leading medical cause of years lived with disability worldwide (GBD 2019 Diseases and Injuries Collaborators 2020). SZ is a severe neuropsychiatric disease that typically emerges in late adolescence, persists throughout adult life, and affects about 1% of the population (Jablensky 1995). Together, the direct and indirect costs of SZ-related psychotic disorders amount to €93.9 billion (Gustavsson et al. 2011). The high hospitalization rates and high levels of disease-related incapacity to work and early retirement lead to a high disease burden (GBD 2019 Diseases and Injuries Collaborators 2020). Furthermore, a substantial proportion of patients (30–50%) experience an unfavourable disease course and residual symptoms, i.e., cognitive impairment and negative symptoms, that remain after acute treatment (Falkai and Schmitt 2022). These symptoms are generally very difficult to treat with psychotherapy or antipsychotics and cause multifaceted disability, including functional impairments in everyday life that prevent successful social and professional reintegration (Nielsen et al. 2015). In SZ, twin studies have reported a heritability estimate of this disorder of about 60–80% (Sullivan et al. 2003). In addition, environmental factors such as obstetric complications, virus infections of the mother and childhood trauma also contribute to an increased risk of the disease (Schmitt et al. 2014).

## The neurodevelopmental hypothesis

**Fig. 1:**
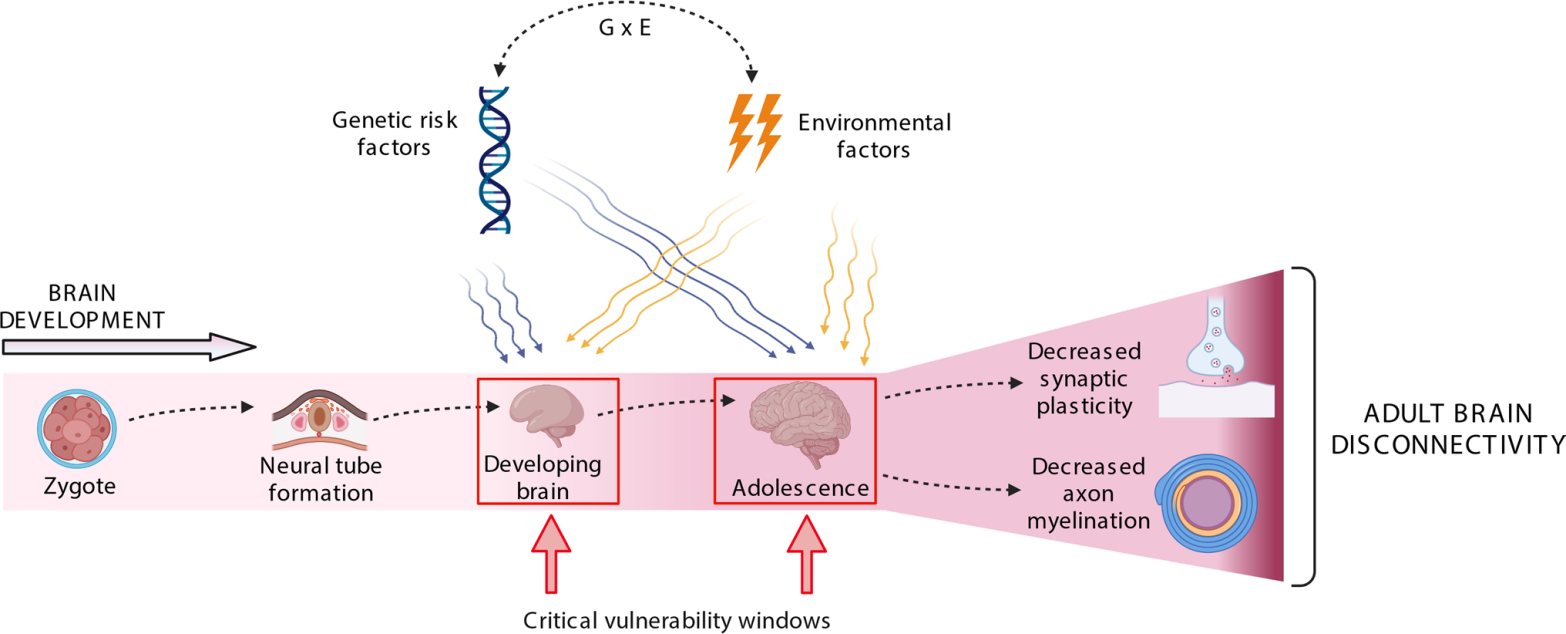
Impact of genetic and environmental factors during neurodevelopment in schizophrenia: two vulnerable periods in brain development are the prenatal period and adolescence. During these critical periods, genetic and environmental risk factors of schizophrenia act together to induce deficits in synaptic plasticity and myelination. As consequence, impaired micro- and macroconnectivity is the basis of cognitive deficits and symptoms of the disease, which arise in young adulthood

It has been proposed that a variety of environmental factors may interact with genetic risk factors during the pre- or postnatal period to induce symptoms of SZ in early adulthood (Schmitt et al. 2014). This neurodevelopmental hypothesis was first introduced in 1986 (Weinberger 1986) and proposes that SZ is related to genetic and environmental adverse conditions leading to abnormal brain development during the perinatal period, whereas symptoms of the disease appear in early adulthood (Weinberger 1996). In SZ, a pre-existing disturbed neuronal network may be triggered towards disease during a vulnerable period in adolescence. Specifically, the second trimester and perinatal period has been implicated in the pathophysiology of SZ (Fatemi and Folsom 2009). Synaptogenesis is established during the second and third trimester of pregnancy and continues during childhood (Hall and Bray 2022). In a proposed "two-hit" model, early perinatal insults (genetic background and/or environmental factors) may lead to dysfunction of neuronal networks and a vulnerable status, while a second "hit" during a critical brain development period in adolescence may induce the onset of the disease (Fig. 1) (Keshavan and Hogarty 1999). During this critical period in adolescence, a synaptic pruning process with excessive elimination of synapses and loss of synaptic plasticity may lead to a disturbed microconnectivity and exacerbation of symptoms in the predisposed brain (Keshavan and Hogarty 1999; Schmitt et al. 2014). Additionally, myelination of the heteromodal association cortex like the prefrontal cortex occurs during this period (Peters et al. 2012) and a deficit in myelination and oligodendrocyte number may contribute to disturbed macroconnectivity in SZ (Hof et al. 2003; Schmitt et al. 2009; Falkai et al. 2016). According to the neurodevelopmental hypothesis, prodromal and symptoms of SZ occur for the first time in adolescence (Häfner 2007). Along the same lines, those subjects who will eventually suffer SZ already show unspecific signs of a slight brain dysfunction before the onset of the disease, manifested as a mild cognitive impairment or subtle motor abnormalities (Cuesta et al. 2018; Kahn 2020).

## SZ-related genetic factors with impact on neurodevelopment

Family, twin, and adoption studies have provided compelling evidence of the contribution of genetic factors to SZ risk, with an estimated heritability (h^2^) around 60 to 80% (Sullivan et al. 2003). These estimates have been confirmed by nation-wide register-based studies (Lichtenstein et al. 2009). Recent large-scale genomics approaches have finally improved our understanding of how specific genetic factors contribute to SZ risk. Genome-wide association studies (GWASs) have provided convincing evidence of the remarkable role that common genetic variants play in the definition of the individual vulnerability background to suffer SZ (Dennison et al. 2020).

The largest GWAS in SZ to date has reported 287 independent genetic risk loci for SZ (Trubetskoy et al. 2022). The results of the study provided biological insight into the biological underpinnings of SZ: the associations were found to be enriched in cortical inhibitory interneurons and excitatory neurons in the cerebral cortex and hippocampus (pyramidal and granule cells), reinforcing the notion that SZ is primarily a neuronal disorder. The cellular components or molecular functions more enriched for these associations were related to neuronal excitability, and synaptic (especially the post-synapse) structure and function. In addition, the enrichment of these associations in biological processes such as nervous system development, regulation of neuron differentiation, or neurogenesis, were of special interest within the framework of the neurodevelopmental hypothesis of SZ. Moreover, the *C4A* locus in the Major Histocompatibility Complex (MHC) in chromosome 6, which is the locus with the top association with SZ, has been shown to participate in synaptic pruning, determination of synapse density, and microglial engulfment of synapses (Sekar et al. 2016; Yilmaz et al. 2021).

Moreover, large-scale genomic studies analysing structural variants (copy number variation, CNV) or rare (low frequency) genetic variation have also provided convergent evidence regarding the impact of SZ genetic risk burden on the synapse (Marshall et al. 2017; Singh et al. 2022). The case of structural variants is especially interesting since several of the CNV loci identified in this study overlap with genomic regions previously implicated with developmental syndromes like autism spectrum disorder (2p16.3 [*NRXN1* gene, Neurexin-1 protein]) (Tromp et al. 2021) or 22q11.2 deletion syndrome (Francisco 2022). However, an important limitation of genetic association studies is that they are not designed to discriminate the developmental stage at which each genetic risk factor contributes to the individual vulnerability background.

Despite this hurdle, a growing body of evidence indicates that some of the genes involved in SZ risk act, at least, during prenatal development (Hall and Bray 2022). *NRXN1*, overlapping with a SZ risk CNV locus (2p16.3), plays a remarkable role in synapse formation at early stages of development (Tromp et al. 2021). Exome sequencing has identified several genes also important during early stages of development for neurite outgrowth and axon/dendrite branching: *SP4* (Transcription Factor Sp4), *SETD1A* (Histone-lysine N-methyltransferase SETD1A), or *TRIO* (Triple functional domain protein) (Singh et al. 2022). Finally, GWAS have identified common risk variants in several genes that also participate in neurite outgrowth and synapse formation: *ZNF804A* (Zinc finger protein 804A), *CNTN4* (Contactin-4), *LRRC4B* (Leucine-rich repeat-containing protein 4B), or *DCC* (Netrin receptor DCC) (Trubetskoy et al. 2022). Several of these genes (*NRXN1*, *TRIO*) play a prominent role in synaptic plasticity throughout development and adult life. In these cases, identifying the developmental timing of their aetiological mechanism using only genetic association studies remains a challenge. A better knowledge of the effects of genetic variation on gene regulation during pre- and postnatal development could help to clarify these questions. In this vein, some studies have integrated the results of GWAS with information on quantitative trait loci (QTLs) known to be active in the foetal brain. These approaches have shown that mechanistic effects of *CNTN4*, *PCDHA7*, *PCDHA8* (Protocadherins alpha-7 and alpha-8), and other genes on synaptic SZ risk are very likely to take place in utero, although posterior postnatal influences cannot be ruled out (Walker et al. 2019; Hall et al. 2021). This is the case of *NRNX1* and *TRIO* genes, which also have clear functions in glutamatergic transmission in the adult hippocampus (Hall and Bray 2022). Such a dual role also can be observed in *CACNA1C* gene, one of the most robust transdiagnostic genetic markers for SZ and bipolar disorder (Mullins et al. 2021; Trubetskoy et al. 2022). *CACNA1C* codes for a voltage-dependent calcium channel, voltage-dependent L-type calcium channel subunit alpha-1C (also known as Cav1.2), with an important role both in synaptic plasticity in adult brain (Nanou and Catterall 2018) and regulation of Ca^2+^ activity and formation of neuronal networks during neurodevelopment (Smedler et al. 2022). Interestingly, a recent post-mortem study in the temporal cortex (BA21) of SZ patients has reported a downregulation of Cav1.2 (*CACNA1C*) and Cav1.3 (*CACNA1D*) mRNAs (Schmitt et al. 2022).

## Induced pluripotent stem cell (IPSC) modelling

Recent advances in stem cell technology, now allowing to obtain iPSCs from blood, skin, or other somatic tissues and their reprogramming into cell types of the central nervous system, holds promise for the understanding of the developmental functional cellular mechanisms that connect genetic with an increased SZ risk at unprecedented resolution (Howes and Shatalina 2022). For example, several recent studies have shown the deleterious effect of deletions and aberrant expression of *NRXN1* gene on neuronal excitability and synaptic function in iPSC-derived neurons (Flaherty et al. 2019; Pak et al. 2021; Avazzadeh et al. 2021). In the case of *ZNF804* gene, IPSC-derived neurons were used to uncover a novel subcellular distribution within somatodendritic compartments and the regulatory function of this molecule in neurite formation and dendritic spine structure (Deans et al. 2017). Another study based on IPSC-derived developing cortical interneurons has shown that several SZ GWAS loci converge on the PKC pathway, leading to an abnormal arborization during development (Liu et al. 2022). The same study, interestingly, observed that in IPSC-derived developing glutamatergic neurons SZ GWAS loci converge on the ion transport pathway, and disruption of one of the members of this pathway, *CACNA1D*, led to alteration in calcium currents in these developing cells (Liu et al. 2022).

Such cellular models have also provided interesting hints to understand the role of classical chromosomal aberrations known to increase risk for schizophrenia. In the case of 22q11.2 deletion, the use of such models has allowed the identification of trans effects of this deletion during the process of neuronal differentiation with a large effect on genes previously identified in SZ GWAS, as for example *MEF2C* (Myocyte-specific enhancer factor 2C) (Nehme et al. 2022). A similar approach based on the 15q13.3 deletion identified a SZ-associated loss of function genetic variant in the *OTUD7A* gene (OTU domain-containing protein 7A), mapping to this region, that led to impaired synapse development in IPSC-derived excitatory neurons (Kozlova et al. 2022).

## Environmental factors contributing to risk of SZ

In SZ, robust evidence indicates that cannabis use, exposure to stressful events during childhood and adulthood, and a history of obstetric complications are well-replicated risk factors (Belbasis et al. 2018). However, these environmental factors, which play a role during the prenatal period and adolescence have also been related to depression, anxiety, autism spectrum disorder and attention deficit hyperactivity disorder (ADHD) (Markham and Koenig 2011; Class et al. 2014). The second trimester of pregnancy and the adolescence are particularly vulnerable brain development periods very sensible to environmental stressors (Fig. 1). Several meta-analyses have shown an association between birth and obstetric complications and SZ. The pooled odds ratio for the exposure to OCs on subsequent development of SZ was 2.0 (Geddes and Lawrie 1995). Obstetric complications include bleeding, preeclampsia, diabetes, rhesus incompatibility, asphyxia, uterine atony, and with the highest risk connected to emergency caesarean section and placental abruption. The foetal abnormalities with the highest effect on SZ risk are low birth weight (OR ~ 3.2), small head circumference (OR ~ 1.6) and congenital malformations (OR ~ 2–2.5) (Waddington et al. 2008; Harper et al. 2015). SZ has been associated with low gestational age at birth with an odds ratio of 3.2 (Hultman et al. 1999). Maternal bleeding during pregnancy has been found to be associated with SZ with an odds ratio of 3.5 (Hultman et al. 1999). Low birth weight is a general marker of disturbances of the intrauterine environment (Fineberg et al. 2013). Another meta-analysis found associations between SZ and different obstetric complications, use of incubator, prematurity and premature rupture of membranes (Geddes et al. 1999). Studies with individuals at high risk for psychosis who converted into SZ showed that they present more obstetric complications compared to non-converting individuals (Mittal et al. 2009). A common factor of these complications is perinatal hypoxia (Zornberg et al. 2000), which in animal models induced SZ-associated behavioural deficits in early adulthood, such as deficits in prepulse inhibition of acoustic startle response (Fendt et al. 2008). A recent meta-analysis revealed that SZ patients with obstetric complications had a poorer verbal and working memory performance than patients without obstetric complications (Amoretti et al. 2022).

Maternal stress during the prenatal period has been shown to be a risk factor of SZ (Markham and Koenig 2011). These risk factors include maternal psychological stress exposure due to e.g. unwantedness of a pregnancy, war experience or natural disaster (Brown 2002; Spauwen et al. 2004). Children of mothers who experienced serious life events such as war experience developed SZ more frequently than expected (van Os and Selten 1998). Prenatal stress is known to influence function of the hypothalamic–pituitary–adrenal (HPA) axis, which is the major stress neuroendocrine system of the body, and the protective capacity of the placenta (Weinstock 2008). Childhood trauma is a severe form of stress, which influences the HPA axis and renders individuals more vulnerable to develop SZ (Popovic et al. 2019). In a meta-analysis of 18 case–control studies, adverse experiences in childhood significantly increased the risk to develop SZ (Varese et al. 2012). Specifically, a strong association between childhood adversity, including trauma, and SZ has been shown with odds ratio between 2 and 3 (Varese et al. 2012). Epidemiological studies show that early stress in the form of abuse and neglect during childhood plays an important role as a risk factor for SZ (Bonoldi et al. 2013). In SZ patients, although the most frequently reported subtype of trauma was emotional neglect, also rates of physical abuse and physical neglect were increased (Larsson et al. 2013). However, childhood trauma is not only a risk factor for SZ, but also for other mental disorders such as affective disorders and ADHD (Popovic et al. 2019).

Potential stress-related factors for SZ are migration and urbanicity. A meta-analysis reported an association with urban environment and SZ (van Os et al. 2010). Individuals living in a higher degree of urbanization had a higher risk to develop SZ than people living in rural areas (Pedersen and Mortensen 2001). In healthy probands, city living was associated with increased amygdala activity, whereas urban upbringing affected the anterior cingulate cortex, and the stress response (Lederbogen et al. 2011). In first- and second-generation migrants as well as in minority groups across all cultures, psychotic symptoms have been shown to be increased (Rapoport et al. 2012) (37). It has been assumed that social status, e.g. occupying a minority position or experiencing social exclusion, promotes the development of SZ (van Os et al. 2010). In addition, maternal malnutrition has been related to the risk of brain defects and neuropsychiatric disorders including SZ (Cortés-Albornoz et al. 2021). Famine periods during second world war and in China doubled the risk for SZ (Susser et al. 1996; Xu et al. 2009). As consequence of malnutrition, deficits in vitamin D, polyunsaturated fatty acids, folic acid, choline, and iron intake have been regarded to play a role in the pathophysiology of the disease (Martinat et al. 2021; Freedman et al. 2021).

Infections during pregnancy activate the maternal immune system and can trigger neuroinflammation of the foetal brain during neurodevelopment. Evidence from animal studies suggests that SZ-related symptoms can be induced by viral infections with e.g. Influenza A and Cytomegalovirus (Elgueta et al. 2022) or perinatal induction of neuroinflammation with e.g. poly I:C, which mimics anti-viral innate immune responses (Ding et al. 2019). Severe neuroinflammation during pregnancy has been linked to preterm births, abortions, and microcephaly (Ganguli and Chavali 2021). In nonhuman primates, maternal immune activation induces cognitive dysfunction and deficits in brain growth, characterized by grey and white matter prefrontal volume deficits in adulthood (Vlasova et al. 2021). Future studies should investigate the impact of SARS-CoV-2 infection during the prenatal period since the consequences of the cytokine storm on brain development are unknown (Figueiredo et al. 2021). In fact, retrospective studies have shown an association between SZ and timing of birth during infectious epidemics induced by influenza, polio, diphtheria and measles (Eyles 2021).

## Impact of neurodevelopmental disturbances on brain connectivity in SZ

One of the first neuroimaging findings in SZ was the enlargement of ventricles (Johnstone et al. 1976). This was followed by magnetic resonance imaging (MRI) studies demonstrating more subtle grey matter volume loss especially in the prefrontal cortex, superior temporal gyrus and cingulate cortex (Qi et al. 2022). A meta-analysis of voxel-based morphometry and diffusion tensor imaging studies revealed widespread white matter alterations including decreased fractional anisotropy in fronto-temporal-limbic pathways (Vitolo et al. 2017). Dysconnectivity in fronto-temporal and limbic regions has been described by resting-state functional MRI studies (Brandl et al. 2019). One of the most replicated structural MRI-based finding in SZ is hippocampal volume reduction with volume loss in all subregions (Haukvik et al. 2018). The neuronal network between the prefrontal cortex and hippocampus is critical for cognitive domains such as working memory and verbal memory (Bähner and Meyer-Lindenberg 2017; Vargas et al. 2018). Such a brain network has been shown to be disturbed in SZ, mainly due to neurodevelopmental disturbances (Bullmore et al. 1997; Peters et al. 2012). Animal models studies provided convergent evidence, since perinatal hippocampal lesions induced dysfunction of the prefrontal cortex in early adulthood of rats, leading to an impaired SZ-related behaviour such as reduced prepulse inhibition of acoustic startle response (Lipska 2004).

In adolescent subjects with clinical high risk for SZ, those who convert to psychosis showed accelerated gray matter reduction in the prefrontal cortex and enlarged ventricles compared with those subjects who did not convert and healthy controls (Cannon et al. 2015). A recent meta-analysis confirmed grey matter volume loss of the right and left superior frontal gyrus in subjects at high risk for psychosis (Ding et al. 2019). Moreover, lower cortical thickness has been observed in a large sample of individuals with clinical high risk for psychosis (ENIGMA Clinical High Risk for Psychosis Working Group et al. 2021). Additionally, patients with adolescent onset of SZ had white matter abnormalities compared to healthy controls, pointing to a neurodevelopmental pathology (Seitz-Holland et al. 2022). During this vulnerable brain period, the prefrontal cortex matures through synaptic pruning and myelination (Huttenlocher 1979; Huttenlocher and Dabholkar 1997; Gogtay et al. 2004). Accordingly, a loss of synaptic elements in the prefrontal cortex has been detected in post-mortem studies in SZ (Berdenis van Berlekom et al. 2020). This loss of microconnectivity can be accompanied by a deficit in myelination, leading to disturbed macroconnectivity. In the prefrontal cortex and hippocampal subregion cornu ammonis 4, a loss of oligodendrocytes, which are the myelinating glia cells of the brain, has been reported in SZ (Hof et al. 2003; Schmitt et al. 2009; Falkai et al. 2016). The loss of hippocampal oligodendrocytes was associated with decreased volumes in the neuronal Papez circuit, pointing to impaired connectivity (Falkai et al. 2020).

Using neuroimaging, environmental factors have been shown to influence connectivity in SZ. For instance, in patients with SZ, childhood trauma was associated with disturbances of white matter integrity and functional connectivity in neuronal networks (Cancel et al. 2019). Animal models have shown that chronic stress results in degeneration of hippocampal neurons and atrophy of dendrites (Sapolsky et al. 1990; Watanabe et al. 1992). In SZ patients and their siblings, foetal hypoxia predicted reduced gray matter volume and increased cerebrospinal fluid, most strongly in the temporal lobe. In SZ patients, prenatal hypoxia correlated also with ventricular enlargement (Cannon et al. 2002). Obstetric complications induce brain abnormalities ranging from decreased grey matter volume and increased ventricles up to reduced hippocampus volume (Costas-Carrera et al. 2020). A reduced hippocampus volume has been reported in SZ patients and controls with obstetric complications (Haukvik et al. 2010). Asphyxia at birth was related to smaller intracranial volume and smaller cortical surface areas in frontal, temporal, insular and parietal regions (Wortinger et al. 2020). In premature infants, perinatal white matter injury is based on hypoxia and is accompanied by neuroinflammation, decreased oligodendrocyte maturation and myelin damage (Motavaf and Piao 2021). Elevated expression of inflammation-related genes and an activation of microglia, the resident immune defenders of the brain, have been detected in post-mortem studies in SZ (van Kesteren et al. 2017). A relationship between premature birth, perinatal hypoxia, white matter deficits with oligodendrocyte damage and an activated immune system has been proposed to underlie the pathophysiology of SZ (Chew et al. 2013; Jenkins 2013). In this context, maternal infection with immune activation during pregnancy has been shown to impair dendritic spine development and to impair synaptic plasticity (Pekala et al. 2021). Reduced synaptic plasticity along with reduced dendritic spines, decreased expression of synaptic genes and abnormal synaptic neurotransmission has been reported in SZ, is related to impaired connectivity (Fig. 1) and results in cognitive deficits (Wu et al. 2022).

Neuroimaging studies have provided interesting evidence of the interplay between SZ genetic risk and brain structure/function leading to behavioural outcomes frequently observed in SZ patients. The analysis of a large cohort of twins analysed the relationship between SZ risk, brain structure and cognitive performance (Toulopoulou et al. 2015). This study showed that at least a fraction of SZ genetic risk is related to an abnormal early development of the brain eventually leading to cognitive deficits. In addition, a recent meta-analysis based on first-degree relatives of SZ patients (therefore carriers of SZ genetic risk) has shown that these individuals present alterations in corticostriatal-thalamic networks, spanning the dorsolateral prefrontal cortex and temporal regions (Cattarinussi et al. 2022b). However, it is not yet clear if brain abnormalities associated with an impaired neurodevelopment in SZ are related just to volume changes or an abnormal connectivity of neuronal networks.

Molecular genetics studies have tried to determine if genetic risk variants in genes with a clear role in neurodevelopment contribute to brain abnormalities observed in SZ patients. Studies using global polygenic risk scores for SZ have shown extensive heterogeneity in the results, with positive or negative correlations with cortical thickness in fronto-temporal areas (Cattarinussi et al. 2022a). Such a lack of specificity might be due to the fact that polygenic risk scores summarize genetic risk irrespective of their possible neurodevelopmental / adult timing. Despite these limitations, some studies based on children cohorts have identified the effect of polygenic risk of SZ with higher global cortical thickness, smaller white matter volumes of the fornix and cingulum, larger medial occipital surface area and smaller surface area of lateral and medial temporal regions (Fernandez-Cabello et al. 2022). Polygenic risk scores based only on genetic variants related to neurodevelopment have so far shown inconclusive results regarding changes in brain structure in non-clinical subjects (Van der Auwera et al. 2017; Spalthoff et al. 2019). However, one of these studies identified an interesting association of *TLE1* gene (Transducin-like enhancer protein 1) with increases of cortical thickness in the upper left temporal gyrus (Spalthoff et al. 2019).

Other studies have analysed the effect of specific genetic variants in genes with a clear role in neurodevelopment using a classical candidate gene design (Gurung and Prata 2015). Among all analysed genes, two of them seem to be the ones with a larger effect on brain connectivity: *CACNA1C* and *ZNF804A*. The evidence of an effect on brain structure/volume of genetic variants in *CACNA1C* is weak due to the lack of convincing replication of original findings (Gurung and Prata 2015). However, literature regarding the functional effects of this gene is more consistent (Guardiola-Ripoll et al. 2022). One of the most replicated findings is the influence of *CACNA1C* genetic variants on the connectivity between the dorsolateral prefrontal cortex and the hippocampus (Paulus et al. 2014). Noteworthy, a very similar effect has been also observed for *ZNF804A* gene (Esslinger et al. 2009, 2011). *CACNA1C* has also been associated with decreased functional connectivity between the right dorsolateral prefrontal cortex and right superior occipital gyrus/cuneus and anterior cingulate cortex (Cosgrove et al. 2017), and reduced activation of the left inferior frontal gyrus (Zhang et al. 2019). With regard to *ZNF804A*, many studies based on resting-state paradigms have shown that genetic variation in this gene has an impact on the positive functional coupling between the left precentral gyrus/inferior frontal gyrus and both the left inferior frontal gyrus, and the left posterior cingulate gyrus (Tecelão et al. 2018). Additionally, the functional connectivity between the hippocampus and the dorsolateral prefrontal cortex is impaired (Zhang et al. 2018). This gene has also been associated with dorsolateral prefrontal cortex coupling with the hippocampus and prefrontal cortex (Rasetti et al. 2011; Zhao et al. 2020; Yang et al. 2021). Finally, a recent study has identified a genetic interaction effect between *CACNA1C* and *ZNF804A* modulating the activity ventral caudate medially and within the left hemisphere, the superior and inferior orbitofrontal gyrus, the superior temporal pole and the ventral-anterior insula during a working memory task (Guardiola-Ripoll et al. 2022).

Other genes that harbour genome-wide associated genetic variants have also been analysed in this context: *TCF4* (Transcription factor 4), *ANK3* (Ankyrin-3), or *NCAN* (Neurocan core protein), among others (Gurung and Prata 2015). However, the evidence of the effect of these genes on connectivity changes with a neurodevelopmental origin is less convincing. Even for *CACNA1C* and *ZNF804A*, although results are more solid, the question remains whether their influence on the aforementioned connectivity parameters has a neurodevelopmental component or involve neural network regulation in the adult brain (or both).

## Interplay between genetic and environmental factors in SZ

Despite compelling evidence of the contribution to risk of genetic and environmental factors to SZ risk, their interplay within a neurodevelopmental framework has not yet been understood. Gene x Environment interactions (G x E) have been hypothesised to play a central role in the differential risk of SZ (van Os et al. 2008). Under this model, the individual genetic background modulates the sensitivity to environmental factors. Several studies have shown that G x E processes are important upon exposure to infections, cannabis use, psychosocial stress, or childhood adversity (Wahbeh and Avramopoulos 2021). However, current evidence suggest that G × E might not be the only process relevant for SZ risk. Other models like GE correlation (Warrier et al. 2021), or even pure additive models with no interaction (Pignon et al. 2022) might also drive the effects of childhood trauma or other sources of psychosocial stress on SZ risk.

## Conclusion

Taken together, the results from genetic and environmental factors highlight the role of synaptic dysfunction an impaired myelination in the pathophysiology of SZ. Synaptic plasticity is a key biological process not only in the adult brain but also in developmental stages of the central nervous system, during the establishment and consolidation of neural networks (Forsyth and Lewis 2017). Here, we show that at least part of the genetic and environmental risk of SZ contributes to neurodevelopmental abnormalities that may lead to vulnerable synaptic networks and impaired myelination in the adult brain. Subsequently, SZ genetic risk may also contribute to active processes interfering with synaptic plasticity in the adult brain. Evidence supports such a dual role of SZ genetic risk throughout brain development and adolescence. Further research is needed to understand i) the timing of the different complex biological processes taking place, and ii) the interplay between genetic and environmental factors during these processes. Here, recent ground-breaking advances in stem cell methodologies may pave the way for the identification of the specific neurodevelopmental mechanisms that increase SZ risk.

## Declarations

### Data availabilitys

Data are not available.
